# Th17/1 and ex-Th17 cells are detected in patients with polyarticular juvenile arthritis and increase following treatment

**DOI:** 10.1186/s12969-024-00965-5

**Published:** 2024-03-02

**Authors:** Stephanie Wood, Justin Branch, Priscilla Vasquez, Marietta M. DeGuzman, Amanda Brown, Anna Carmela Sagcal-Gironella, Saimun Singla, Andrea Ramirez, Tiphanie P. Vogel

**Affiliations:** 1 Division of Rheumatology, Department of Pediatrics, Baylor College of Medicine and Texas Children’s Hospital, 1102 Bates Street Suite 330, Houston, TX 77030 USA; 2 Center for Human Immunobiology, Texas Children’s Hospital, 1102 Bates Street Suite 330, Houston, TX 77030 USA

**Keywords:** IL-6, STAT3, Th17, Treg, Th17/1, Ex-Th17, Polyarticular juvenile idiopathic arthritis

## Abstract

**Background:**

A better understanding of the pathogenesis of polyarticular juvenile idiopathic arthritis (polyJIA) is needed to aide in the development of data-driven approaches to guide selection between therapeutic options. One inflammatory pathway of interest is JAK-STAT signaling. STAT3 is a transcription factor critical to the differentiation of inflammatory T helper 17 cells (Th17s). Previous studies have demonstrated increased STAT3 activation in adult patients with rheumatoid arthritis, but less is known about STAT3 activation in polyJIA. We hypothesized that Th17 cells and STAT3 activation would be increased in treatment-naïve polyJIA patients compared to pediatric controls.

**Methods:**

Blood from 17 patients with polyJIA was collected at initial diagnosis and again if remission was achieved (post-treatment). Pediatric healthy controls were also collected. Peripheral blood mononuclear cells were isolated and CD4 + T cell subsets and STAT activation (phosphorylation) were evaluated using flow cytometry. Data were analyzed using Mann–Whitney U and Wilcoxon matched-pairs signed rank tests.

**Results:**

Treatment-naïve polyJIA patients had increased Th17 cells (CD3 + CD4 + interleukin(IL)-17 +) compared to controls (0.15% v 0.44%, *p* < 0.05), but Tregs (CD3 + CD4 + CD25 + FOXP3 +) from patients did not differ from controls. Changes in STAT3 phosphorylation in CD4 + T cells following ex vivo stimulation were not significantly different in patients compared to controls. We identified dual IL-17 + and interferon (IFN)γ + expressing CD4 + T cells in patients, but not controls. Further, both Th17/1 s (CCR6 + CD161 + IFNγ + IL-17 +) and ex-Th17s (CCR6 + CD161 + IFNγ + IL-17^neg^) were increased in patients’ post-treatment (Th17/1: 0.3% v 0.07%, *p* < 0.05 and ex-Th17s: 2.3% v 1.4%, *p* < 0.05). The patients with the highest IL-17 expressing cells post-treatment remained therapy-bound.

**Conclusions:**

Patients with polyJIA have increased baseline Th17 cells, potentially reflecting higher tonic STAT3 activation in vivo. These quantifiable immune markers may identify patients that would benefit upfront from pathway-focused biologic therapies. Our data also suggest that inflammatory CD4 + T cell subsets not detected in controls but increased in post-treatment samples should be further evaluated as a tool to stratify patients in remission on medication. Future work will explore these proposed diagnostic and prognostic biomarkers.

**Supplementary Information:**

The online version contains supplementary material available at 10.1186/s12969-024-00965-5.

## Background

Juvenile idiopathic arthritis (JIA) is the most common childhood rheumatic disease affecting approximately 1:1000 children [1]. The polyarticular JIA (polyJIA) subtype is the most clinically similar to adult rheumatoid arthritis (RA), yet its pathogenesis remains poorly understood. Untreated, JIA can cause pain, lead to joint damage, and result in disability. Fortunately, therapeutics for JIA have greatly expanded, and there are now options that span a variety of mechanisms. This has made inactive disease the treatment goal for all patients [2]. Data and expert consensus support achieving the goal of inactive disease, regardless of the specific choice of medication(s) or their mechanism [3]. While many patients respond promptly and well to the initiation of treatment, this approach can, and does, lead to instances of failure of a cascade of empirically selected agents prior to arriving at the therapeutic that allows for development of clinically inactive disease. However, there are currently insufficient data to guide selection amongst therapeutic options in a directed manner [4]. Identification of biomarkers that could be detected at diagnosis, distinguish molecularly between subsets of patients with clinically similar disease, and allow for targeted therapeutic recommendations is the apex of arthritis treatment.

One targetable inflammatory pathway of interest that might stratify patients into molecular groups is the interleukin(IL)-6/signal transducer and activator of transcription 3 (STAT3) pathway, for which an anti-cytokine pathway biologic therapy has been approved for polyJIA [5]. IL-6/IL-6 receptor (R) engagement and activation of gp130 signaling leads to Janus kinase (JAK)-mediated activation of STAT3 [6]. The importance of this pathway in human immunobiology has been clearly demonstrated by rare inborn errors of immunity, wherein defects of IL-6/STAT3 signaling result in phenotypically similar primary immune deficiencies [7], and gain-of-function leads to early-onset, multiorgan autoimmunity, including inflammatory arthritis indistinguishable from JIA [8]. Detection of elevated IL-6 in the serum of patients with RA [9, 10] was an important initial observation on the way to the development of IL-6/IL-6R inhibition [11]. Increases in STAT3 and activated (phosphorylated) STAT3 are correspondingly reported in peripheral blood and synovial fluid of patients with RA at baseline [12, 13]. IL-6 is also elevated in the serum of patients with polyJIA [14], although similar STAT3 studies have not been reported.

The IL-6/STAT3 inflammatory pathway is an important factor in the differentiation of different subsets from naïve CD4 + T cells [11]. IL-6 driven STAT3 activation influences the development of T helper 17 (Th17) cells [15], at the expense of T regulatory (Treg) cell development. Th17 cells are key for host defense against extracellular fungi and play an important role in immune homeostasis, the understanding of the depth of which is continually evolving [16]. Despite their beneficial role, Th17 cells are also a contributing factor in chronic inflammation, including in RA and JIA [17, 18]. The balance of pro- and anti-inflammatory T cells can be influenced by targeted IL-6 pathway blockade [19–22], even in the setting of genetically driven IL-6/STAT3 pathway activation [23].

Detection of polyJIA patients with enhanced IL-6/STAT3 pathway activation may distinguish a subset for whom application of directed biologic therapy may provide targeted treatment. We aimed to profile STAT3 activation and T cell subsets in polyJIA patients before and during treatment.

## Methods

### Patients and samples

Patients were included if they were diagnosed with active polyarticular juvenile idiopathic arthritis [24], regardless of seropositivity, and were treatment-naïve. Patients were considered treatment-naïve if they had not received any prescribed therapy for at least 3 months prior to enrollment; patients receiving over-the-counter non-steroidal anti-inflammatories were not excluded. Peripheral blood samples were collected from patients at the time of enrollment and, whenever possible, after achieving remission [25]. Peripheral blood mononuclear cells (PBMC) were isolated from samples collected in anticoagulant tubes using density gradient centrifugation (Ficoll Paque Plus, Sigma) and cryopreserved for batched analysis.

### Flow cytometry

#### T helper 17 cell staining

Up to 1.2 × 10^6 reanimated PBMCs were stimulated overnight (14 h) using phorbol 12-myristate 13-acetate (PMA, 50 ng/mL) and calcimycin (500 ng/mL) in the presence of brefeldin A at 1:1000 dilution (GolgiPlug, BD Biosciences). Stimulated cells were first stained using fixable viability dye (Zombie Yellow, Biolegend) and surface markers for 25 min at room temperature followed by a PBS wash, then fixed and permeabilized (Cytofix/Cytoperm, BD Biosciences) on ice for 20 min. Permeabilized cells were then stained for 1 h at room temperature with intracellular antibodies.

#### T regulatory cell staining

Up to 1.2 × 10^6 reanimated PBMC were stained first for surface markers for 20 min at room temperature, then fixed and permeabilized using the Foxp3/Transcription Factor Staining Buffer Set (eBioscience), per manufacturer’s instructions. Permeabilized cells were then stained with intracellular antibodies for 30 min at room temperature.

#### Phospho-STAT staining

Up to 1.2 × 10^6 reanimated PBMC were either left unstimulated or stimulated with IL-6 (10 ng/mL), IFNα (50 ng/mL), or IL-27 (10 ng/mL) for 30 min. Cells were fixed for 10 min at 37 °C (Cytofix, BD Biosciences) and permeabilized for 30 min on ice (Perm Buffer III, BD Biosciences), then stained for both surface markers and intracellular molecules for 1 h at room temperature.

#### T cell plasticity staining

Up to 1.2 × 10^6 reanimated patient PBMCs were stimulated overnight (14 h) with PMA (50ng/mL) and calcimycin (500 ng/mL) in the presence of brefeldin A at 1:1000 dilution (GolgiPlug, BD Biosciences). Stimulated cells were first stained using fixable viability dye (Zombie Yellow, Biolegend) and surface markers for 20 min at room temperature followed by a PBS wash, then fixed and permeabilized (Foxp3/Transcription Factor Staining Buffer Set (eBioscience), per manufacturer’s instructions. Permeabilized cells were then stained with intracellular antibodies for 30 min at room temperature.

A table of antibodies used for flow cytometry is included in Supp. Methods. Cells were filtered before analysis on a BD LSRFortessa. Data were collected using BD FACSDiva and analyzed using FlowJo v10.6.

### Statistical analysis

Data was analyzed using GraphPad Prism. Mann–Whitney U test was used to compare groups. Paired samples were analyzed using Wilcoxon matched-pairs signed rank test. *P* < 0.05 was considered significant.

## Results

### Patients

Treatment-naïve samples were collected from 17 patients with polyJIA [24] with active disease receiving no more than over-the-counter anti-inflammatory treatment. All subjects had new-onset disease except for one, who was enrolled at the time of a flare three months after discontinuation of prior therapy. Characteristics of the patients are described in Table 1. A post-treatment sample was also collected from 10 patients who had achieved remission [25]. Therapeutic recommendations, including specific agents and time of treatment discontinuation, were made by each patient’s primary rheumatologist, and were not influenced by participation in the study. Two patients received methotrexate only, 3 patients a biologic only, and 5 patients both methotrexate and a biologic (Table I). A time course of events including sample collection(s), remission, medication discontinuation and follow-up are represented in Supplementary Figure 1. Pediatric healthy controls (PC) were recruited from siblings of patients in rheumatology clinic without clinically apparent inflammatory disease.

**Table 1:** Patient characteristics (*n* = 17^a^)

**Demographics**	
	**Mean (range)**
Age, years	10.6 (3-17^b^)
	**#** **(%)**
**Sex**	
Male: Female	4 (24):13 (76)
**Race**	
White	14 (82)
Black	3 (18)
**Ethnicity**	
Hispanic	9 (53)
Non-Hispanic	8 (47)
**Laboratory Findings**	
	**#** **(%)**
RF^c^ (*n* = 16)	4 (25)
Anti-CCP (*n* = 14)	3^d^ (21)
ANA	9 (53)
HLA B27 (*n* = 11)	0
ESR	5 (29)
CRP (*n* = 14)	7 (50)
**Treatment (*n* = 10**^e^**)**	
	**#** **(%)**
MTX only	2 (20)
Biologic only	3 (30)
Both	5 (50)

^a^unless otherwise stated

^b^at sample collection

^c^RF, rheumatoid factor; CCP, cyclic citrullinated peptide; ANA, antinuclear antibodies; HLA B27, human leukocyte antigen B27; ESR, erythrocyte sedimentation rate; CRP, C reactive protein; MTX, methotrexate

^d^all CCP + individuals were also RF +

^e^with pre- and post-treatment samples and clinical remission

### T helper 17 cells

**Fig. 1:**
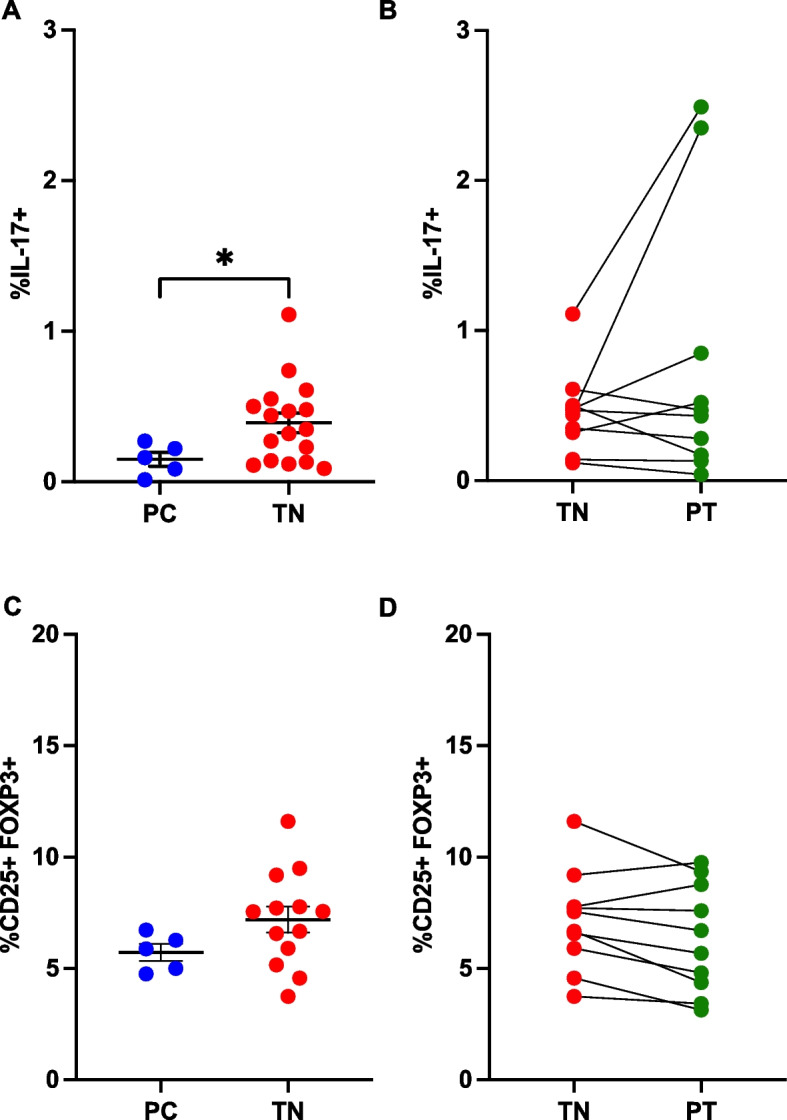
Treatment-naïve polyJIA patients have altered CD4 + T cell subsets. **A** PBMCs from healthy pediatric controls (PC) and treatment-naïve polyJIA patients (TN) were analyzed for IL-17 producing CD3 + CD4 + (Th17) T cells after overnight stimulation with PMA + calcimycin. Treatment-naïve patients had more Th17 cells (PC 0.15 ± 0.05% v TN 0.4 ± 0.07%, *p < 0.05, Mann–Whitney U test). **B** Samples from polyJIA patients that had achieved remission on medication post-treatment (PT) were also analyzed for Th17 cells and compared to the treatment-naïve frequencies for that same patient without significant differences, but with two notable outliers (Wilcoxon matched-pairs signed rank test). **C** T regulatory cells (Tregs = CD3 + CD4 + CD25 + FOXP3 +) identified ex vivo from treatment-naïve patients were not different from healthy pediatric controls (PC 6.3 ± 0.8% v TN 7.1 ± 0.7%, Mann–Whitney U test). **D** Paired analysis of patients’ treatment-naïve and post-treatment samples did not reveal differences in Treg frequencies after treatment (Wilcoxon matched-pairs signed rank test). Black bars represent mean ± SEM. PBMCs, peripheral blood mononuclear cells; IL, interleukin; PMA, phorbol 12-myristate 13-acetate

Th17 cells [26] were identified as CD3 + CD4 + IL-17 + T cells (Supplementary Figure 2). Significantly higher frequencies of Th17 cells were detected in the peripheral blood of treatment-naïve polyJIA patients (0.4 ± 0.07%) compared to pediatric controls (0.15 ± 0.05%, *p* < 0.05, Fig. 1A). The frequencies of Th17 cells in matched treatment-naïve and post-treatment samples from polyJIA patients were overall similar (Fig. 1B), with two notable exceptions whose Th17 cells markedly increased. CD4 + T cells from polyJIA patients did not show significant differences in production of other inflammatory cytokines compared to pediatric controls (Supplementary Figure 3). Further characterization within the Th17 subset revealed no differences in expression of pro-inflammatory cytokines associated with the Th17 lineage (IL-21, IL-22) [27], or in TNFα or interferon(IFN)γ production (Supplementary Figure 4) between polyJIA patients and pediatric controls.

### T regulatory cells

The frequencies of CD3 + CD4 + CD25 + FOXP3 + Tregs [28, 29] in the peripheral blood of patients with polyJIA were also determined (Supplementary Figure 5). No significant differences in Treg frequencies between treatment-naïve polyJIA patients and pediatric controls were detected (Fig. 1C), nor were there significant changes in Treg frequencies in polyJIA patients post-treatment (Fig. 1D). Within the Treg compartment we did not observe differences in expression of markers associated with Treg suppressive function (HELIOS, CD39, and CD73) [30–32] between polyJIA patients and pediatric controls (Supplementary Figure 6).

### STAT activation analysis

**Fig. 2:**
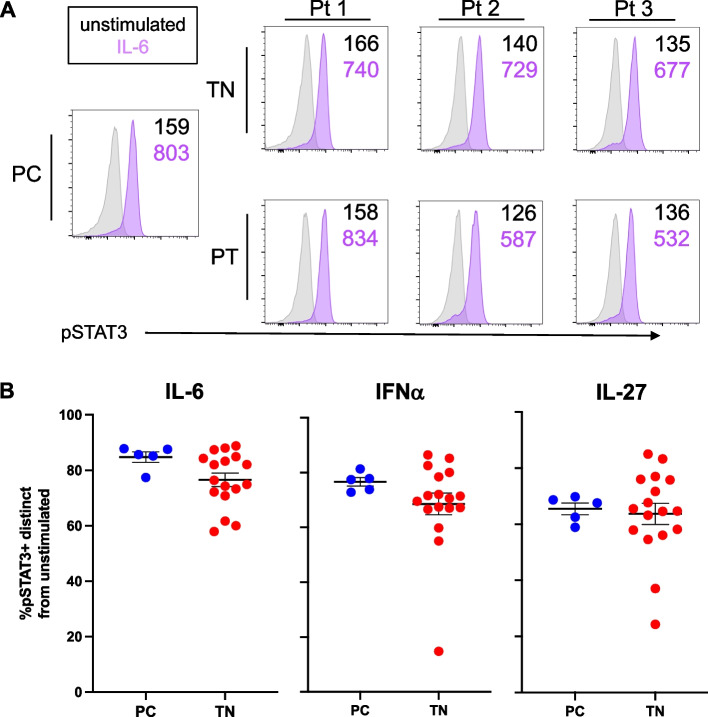
STAT3 activation after ex vivo stimulation of CD4 + T cells from polyJIA patients. **A** PBMCs were left unstimulated (gray) or stimulated ex vivo for 30 min with IL-6 (purple), then activated/phosphorylated (p)STAT3 measured using flow cytometry. Histograms depict representative results from one experiment showing pSTAT3 in CD3 + CD4 + T cells from a healthy pediatric control (PC) and both treatment-naïve (TN) and post-treatment (PT) samples from 3 polyJIA patients (Pt 1–3). Black numbers represent the MFI of pSTAT3 in unstimulated cells and the purple numbers the MFI after IL-6 stimulation. **B** Samples were treated as in (**A**). Data represent the %pSTAT3 + cells in a stimulated sample distinct from when unstimulated, generated using Overton subtraction. Cells from treatment-naïve patients demonstrate less ex vivo response to cytokine stimuli than those from healthy pediatric controls, although not achieving significance (Mann–Whitney U test). Black bars represent mean ± SEM. IL-6: PC 85 ± 1.9% v TN 76 ± 2.4%, IFNα: PC 77 ± 1.5% v TN 69 ± 3.9%, and IL-27: PC 66 ± 2.1% v TN 64 ± 3.8%. PBMCs, peripheral blood mononuclear cells; IL, interleukin; MFI, mean florescence intensity; IFN, interferon

To determine the activation status (i.e. phosphorylation) [6] of STAT3 in CD4 + T cells from patients with polyJIA, peripheral blood samples were stimulated with IL-6, an inflammatory cytokine that activates STAT3 through a gp130-dependent receptor, IFNα, an inflammatory cytokine that activates STAT3 independent of gp130, and IL-27, which also signals using gp130, but is considered to have anti-inflammatory effects (Supplementary Figure 7). Cytokine stimulation resulted in phosphorylation of STAT3 (pSTAT3) compared to unstimulated cells, but neither increased baseline (unstimulated) pSTAT3 nor hyper-phosphorylation of STAT3 following stimulation was detected in CD4 + T cells from polyJIA patients compared to pediatric controls (Fig. 2A). Further, in pooled analysis of all samples, the increase in newly activated (phosphorylated) STAT3 in stimulated samples compared to unstimulated samples, determined using Overton subtraction [33], trended lower in polyJIA patients compared to pediatric controls following inflammatory cytokine stimulation, but was not significant (Fig. 2B). Decreases in ex vivo activation of STAT1 and STAT5 following inflammatory cytokine stimulation were also not significant (Supplementary Figure 8).

### T cell plasticity

**Fig. 3:**
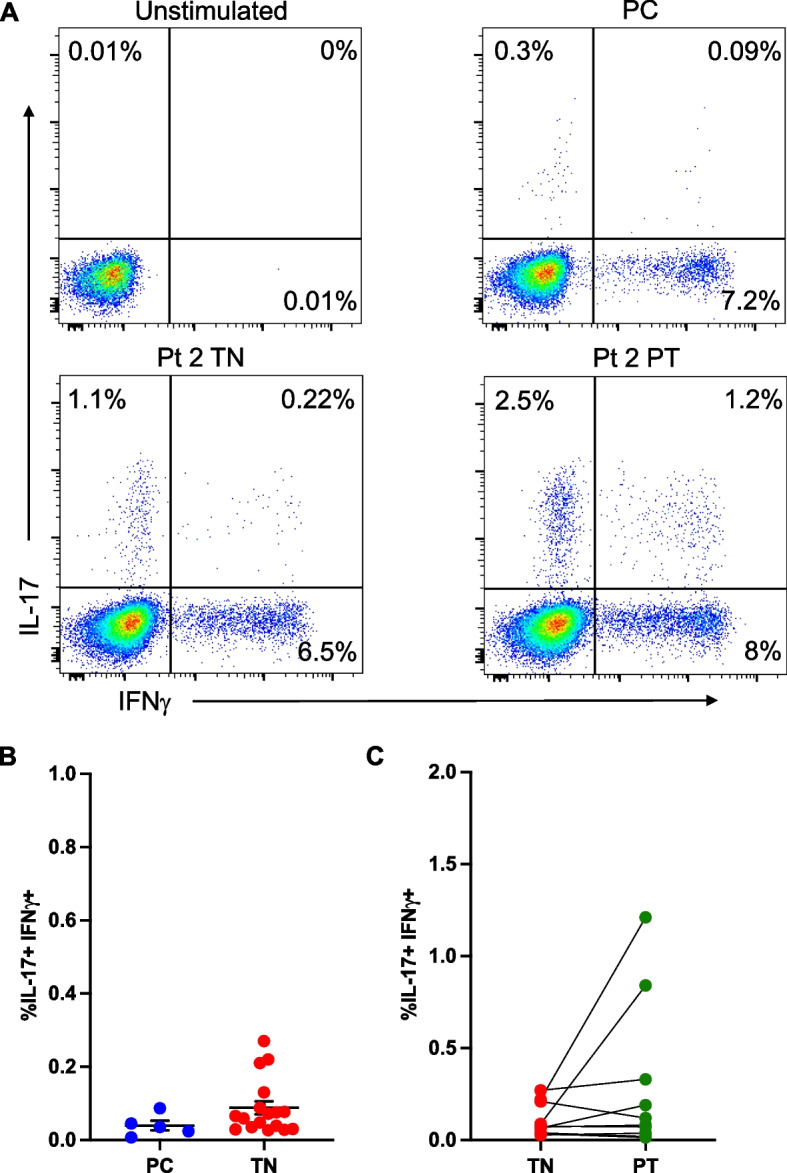
Dual-cytokine expressing CD4 + T cells are detected in a subset of polyJIA patients. **A** PBMCs were left unstimulated or stimulated overnight with PMA + calcimycin and CD3 + CD4 + T cells analyzed for cytokine production. Representative examples from an unstimulated sample and stimulated samples from a healthy pediatric control (PC) and a polyJIA patient (Pt 2), both treatment-naïve (TN) and post-treatment (PT), are shown. Percentages of CD3 + CD4 + T cells producing IL-17 or IFNγ or both are represented in the quadrants. **B** Samples were treated as in (A). Dual IL-17 and IFNγ expressing CD4 + T cells can be detected in a subset of treatment-naïve polyJIA patients, but not healthy pediatric controls (PC 0.04 ± 0.01% v TN 0.09 ± 0.02%, Mann–Whitney U test). **C** Paired analysis of patients’ treatment-naïve and post-treatment samples revealed that some of the outlying IL-17 expressing CD4 + T cells in post-treatment samples also co-express IFNγ (Wilcoxon matched-pairs signed rank test). Black bars represent mean ± SEM. PBMCs, peripheral blood mononuclear cells, PMA, phorbol 12-myristate 13-acetate; IL, interleukin; IFN, interferon

**Fig. 4:**
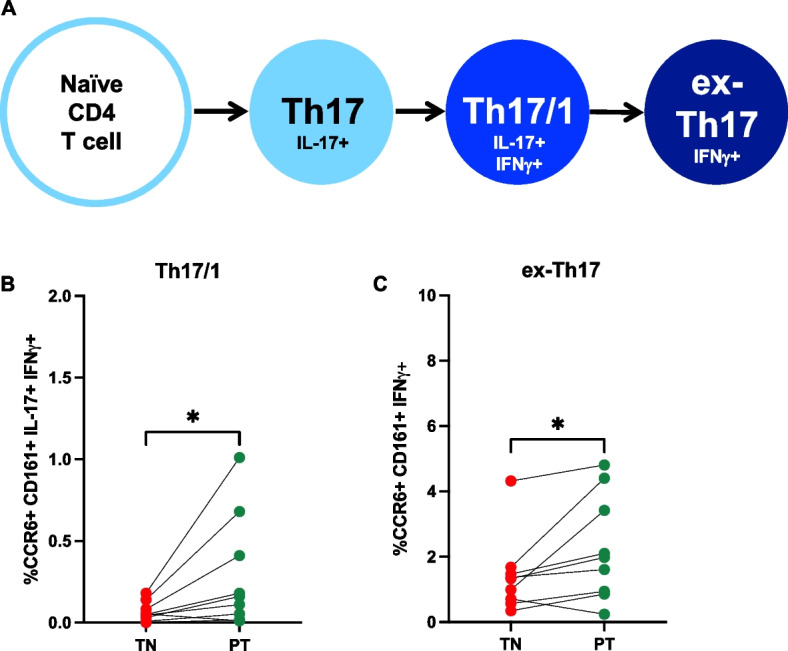
Evidence of altered CD4 + T cell plasticity in post-treatment samples from patients with polyJIA. **A** Naïve CD4 + T cells differentiate into numerous effector subsets and can become Th17 cells implicated in autoimmunity. One model of CD4 + T cell plasticity proposes transdifferentiation of homeostatic Th17 cells into pathogenic IFNγ-expressing Th17/1 cells or ex-Th17 cells. PBMCs were left unstimulated or stimulated overnight with PMA + calcimycin and CD3 + CD4 + T cells analyzed for cytokine production. Cells were additionally surface stained with CCR6 and CD161 to identify transitioned cells. **B** Treatment-naïve (TN) polyJIA patients had detectable Th17/1 cells, but Th17/1 cells were significantly higher in post-treatment (PT) samples (TN 0.07 ± 0.02% v PT 0.3 ± 0.1%, *p < 0.05, Wilcoxon matched-pairs signed rank test). **C** There were more previously IL-17 expressing ex-Th17 cells in samples from post-treatment polyJIA patients than in the same patients when treatment-naïve (TN 1.4 ± 0.4% v PT 2.3 ± 0.5%, *p < 0.05, Wilcoxon matched-pairs signed rank test). Black bars represent mean ± SEM. PBMCs, peripheral blood mononuclear cells, PMA, phorbol 12-myristate 13-acetate; IFN, interferon, IL, interleukin

A population of dual-cytokine expressing (IL-17 + IFNγ +) CD4 + T cells not present in pediatric controls could be detected in samples from patients with polyJIA (Fig. 3A). Though the overall frequencies of these IL-17 + IFNγ + cells were not significantly increased in polyJIA patients compared to pediatric controls (Fig. 3B), it was noted that these cells sharply increased in post-treatment samples from two patients (Fig. 3C). These were the same two patients noted to have elevated post-treatment Th17 cells (Fig. 1B). Transdifferentiation of Th17 cells into Th17/1 cells, expressing both IL-17 and IFNγ, or ex-Th17 cells, producing IFNγ but no longer producing IL-17, is implicated in the acquisition of Th17 pathogenicity found in association with inflammatory diseases [16] (Fig. 4A). Given the finding of dual-cytokine expressing CD4 + T cells in some polyJIA patients, we further investigated T cell plasticity. Th17/1 and ex-Th17 cells were distinguished from Th17 cells in peripheral blood samples from patients with polyJIA by their expression of CCR6 and CD161 [18] (Supplementary Figure 9). Th17/1 cells (Fig. 4B) and ex-Th17 cells (Fig. 4C) were detectable in polyJIA patient samples. Both Th17/1 cells (treatment-naïve: 0.07 ± 0.02% v post-treatment 0.3 ± 0.1%, *p* < 0.05) and ex-Th17 cells (treatment-naïve 1.4 ± 0.4% v post-treatment 2.3 ± 0.5%, *p* < 0.05) were significantly increased in post-treatment samples. The two patients with increased post-treatment Th17 cells (Fig. 1B) and dual-cytokine expressing CD4 + T cells (Fig. 3A) also had the highest frequencies of Th17/1 cells (Fig. 4B). These two individuals (both female, aged 4 (seronegative) and 14 (RF + CCP +) years at diagnosis) achieved remission, but, remained therapy-bound across > 4 years of follow-up, despite multiple attempts at therapy discontinuation.

## Discussion

Activity of the IL-6/STAT3 inflammatory pathway may be able to stratify patients with polyJIA and distinguish a subset for which targeted anti-cytokine therapy is indicated. We evaluated peripheral blood from treatment-naïve polyJIA patients and found significantly elevated frequencies of Th17 cells compared to healthy pediatric controls; Treg cell frequencies did not differ. We also detected a decreased response to ex vivo inflammatory cytokine stimulation in PMBCs from treatment-naïve patients compared to controls, although this trend was not significant. Finally, evaluation of samples collected on therapy after clinical remission revealed, on average, significantly elevated post-treatment frequencies of both Th17/1 and ex-Th17 cells in polyJIA patients compared to their matched treatment-naïve samples. Elevations in post-treatment Th17, Th17/1 and ex-Th17 cells were notable in two patients who remain therapy-bound years after remission.

Investigators have reported elevated Treg cells in the synovial fluid of patients with oligoarticular JIA, not concurrently found in the peripheral blood [34–37]. But, whether patients with polyJIA, particularly treatment-naïve patients, have altered peripheral Treg cells compared to pediatric controls has not been clear [38–41]. We expected to find reduced frequencies of Treg cells, identified by CD25 and FOXP3, in peripheral samples from treatment-naïve polyJIA patients compared to pediatric controls, or between patients from their treatment-naïve to post-treatment samples, but we found no differences (Fig. 1). While Treg cells are classically identified by the expression of FOXP3, there are subsets of cells among FOXP3-expressing T cells, including cells that have developed inflammatory characteristics [42, 43]. It was our goal to identify peripheral Treg cells in order to subset polyJIA patients, however, to identify and track peripheral cells which are genuinely regulatory will likely require additional stratification, if not functional evaluation. These types of evaluations are important to dissect pathogenesis, but less feasible as a real-time evaluation tool.

Peripheral Th17 cells have been evaluated in various cohorts of patients with JIA compared to pediatric controls. Elevated peripheral Th17 cells were detected in systemic-onset JIA and in mixed JIA populations (oligoarticular and polyJIA) [41, 44–46], even though samples were collected during different levels of disease activity and on a spectrum of therapies. However, other investigations reported no differences in peripheral Th17 cells in oligoarticular JIA [47], enthesitis-related arthritis [48] and other mixed JIA cohorts [37, 49, 50]. With respect to the local inflammatory environment, the preponderance of available evidence suggests that the synovial compartment of patients with JIA is enriched for Th17 cells when directly compared to the peripheral blood of the same individuals [37, 41, 47–49, 51]. Depending on a variety of uncontrollable clinical factors, intra-articular sampling for diagnostics/therapeutics is not routinely done at the time of diagnosis of JIA, and rarely in patients with polyJIA. Since synovial fluid is not reliably available for analysis to guide therapeutic recommendations, we determined the peripheral levels of Th17 cells in a large, treatment-naïve poly JIA cohort of 17 patients (Fig. 1), and found, on the whole, Th17 cells to be significantly elevated. Further, there is a spectrum of frequencies of Th17 cells amongst treatment-naïve poly-JIA patients. This may provide an opportunity to distinguish patients with the strongest evidence of IL-6/STAT3 activation, to select anti-IL-6R therapeutics as a tailored approach to treatment.

One easily detected measure of STAT3 activation is phosphorylation at tyrosine 705. We assessed ex vivo STAT3 activation in CD4 + T cells using this parameter. We found that patients with polyJIA had a trend toward decreased newly activated STAT3, particularly after IL-6 stimulation, but this finding was not significant (Fig. 2). Others have also found no difference in phosphorylated STAT3 after IL-6 stimulation in samples from polyJIA patients compared to controls [52]. Interestingly, samples from patients with the rare inborn error of immunity STAT3 gain-of-function, the result of genetic mutations in STAT3 that lead to higher transcriptional capacity than wild-type STAT3, also have decreased phosphorylation of STAT3 after ex vivo inflammatory cytokine stimulation [23]. Since inflamed patients with polyJIA would be expected to have higher activation of the IL-6/STAT3 inflammatory pathway, we had expected to see increases and were surprised by our finding, although this has also been reported in patients with RA [12, 53]. Decreases in newly phosphorylated STAT3 after stimulation may actually reflect higher baseline activation of STAT3 preserved in treatment-naïve samples. Further, decreased responsiveness of patient CD4 + T cells to ex vivo stimulus may result from compensatory mechanisms due to tonic STAT3 activation in vivo [54]. Additionally, as this is only one measure of STAT3 activation, it may be that other ways to measure activation may reveal increases. However, the idea that the IL-6/STAT3 inflammatory pathway is at a higher state in vivo in our patients with polyJIA is support by the findings of increased frequencies of Th17 cells (Fig. 1).

It is increasingly clear that CD4 + T cells marked by expression of IL-17 are a heterogeneous population with both homeostatic and inflammatory properties [16]. However, the true complexity of the roles of IL-17 expressing CD4 + T cells in human autoimmune disease, both in the blood and at local site of inflammation, are still being unraveled. The plasticity of Th17 cells results in the shifting of classic Th17 cells into dual-cytokine expressing cells (IL-17 + IFNγ +), termed Th17/1 cells, as well as ex-Th17 cells, which no longer express IL-17, but retain markers that identify them as prior Th17 cells [18]. How these subtypes may function in the pathogenesis of JIA is not known, as little work thus far has evaluated these subtypes in JIA patients. In one report, investigators found that ex-Th17 cells were not elevated in the peripheral blood of patients with oligoarticular JIA compared to controls, but were elevated in synovial fluid compared to peripheral blood of these patients [47]. Others reported elevated Th17/1 cells in the synovial fluid of patients with JIA [55]. Noting the presence of dual-cytokine expressing CD4 + T cells in polyJIA patients not really detectable in controls (Fig. 3), lead us to evaluate further for Th17/1 and ex-Th17 cells in patient samples. We found that frequencies of both Th17/1 and ex-Th17 cells increased in samples from patients on treatment following remission compared to their treatment-naïve baseline (Fig. 4). This finding is contrary to prior work reporting decreases in ex-Th17 cells in patients with oligoarticular JIA on treatment [56]. This discrepancy might be explained by differences in the inherent disease processes (peripheral analysis in oligoarticular versus polyJIA) or the methodology of the analysis, as the prior work compared different treatment-naïve and post-treatment individuals, but we were able to compare the changes in ex-Th17 cell frequencies in matched treatment-naïve and post-treatment samples from the same patients. The presence of circulating and intra-articular Th17/1 and ex-Th17 cells must be evaluated in additional cohorts of patients with JIA.

It was notable to us that two polyJIA patients had markedly high elevations of post-treatment IL-17 secreting cells: Th17 cells (Fig. 1B), dual-cytokine expressing cells (Fig. 3C), and Th17/1 cells (Fig. 4B). Review of the clinical course of these individuals compared to the other patients in the polyJIA cohort revealed that they have remained therapy-bound > 4 years after attaining clinical remission on medication, by which we mean repeated attempts to wean their medications have led to prompt return of arthritis (Supplementary Figure 1). Current guidelines for treatment of polyJIA do not include recommendations for treatment withdrawal [57], as there is insufficient evidence that clinical parameters can guide these decisions and effective biomarker-based strategies have not yet been developed [58–60]. A subset of patients with JIA are likely destined to remain on treatment for best outcomes, but sustained, drug-free, inactive disease is possible for some [61]. The capacity to use a biomarker to distinguish between these subsets of patients would be a remarkable addition to the treatment arsenal of pediatric rheumatologists. We are interested in further pursuing the idea that a detectable peripheral rise in IL-17 expressing CD4 + T cells, either as Th17 cells or as a shift into dual-cytokine expressing Th17/1 cells, marks a subset of polyJIA patients who will remain therapy-bound in order to retain a clinically inactive disease state.

Our study has several limitations. It is a single-institution study of a small cohort of patients with one subtype of JIA, although it is a large cohort of treatment-naïve samples. The study was purely observational, as we had no influence on the recommendations for treatment or withdrawal made by the patients’ primary rheumatologists. Additionally, study of cells circulating in the periphery of patients does not address the tissue inflammatory environment, but it does allow for serial assessments in an accessible sample type.

## Conclusion

We detected elevated frequencies of Th17 cells in peripheral blood samples from patients with treatment-naïve polyJIA compared to pediatric controls, reflective of activation of the IL-6/STAT3 inflammatory pathway in these patients. In matched samples from the patients following remission post-treatment, frequencies of Th17/1 and ex-Th17 cells were increased compared to the treatment-naïve baselines. Our findings present the opportunity to explore peripheral blood Th17, Th17/1 and ex-Th17 cells as potential biomarkers to guide selection of therapeutic agents and treatment withdrawal decisions.

### Supplementary Information

**Supplementary Materials 1.**

**Supplementary Materials 2.**

## Data Availability

All data generated during this study are included in this published article and its Supplementary material.

## Abbreviations

IFN: Interferon
IL: Interleukin
JAK: Janus kinase
MFI: Mean florescence intensity
PBMCs: Peripheral blood mononuclear cells
PC: Pediatric controls
PMA: Phorbol 12-myristate 13-acetate
PolyJIA: Polyarticular juvenile idiopathic arthritis
PT: Post-treatment
R: Receptor
RA: Rheumatoid arthritis
STAT: Signal transducer and activator of transcription
Th17: T helper 17
TN: Treatment-naïve
TNF: Tumor necrosis factor
Treg: T regulatory

## References

[1] Prakken B, Albani S, Martini A (2011). Juvenile idiopathic arthritis. The Lancet.

[2] Ravelli A, Consolaro A, Horneff G, Laxer RM, Lovell DJ, Wulffraat NM (2018). Treating juvenile idiopathic arthritis to target: recommendations of an international task force. Ann Rheum Dis..

[3] Mannion ML, Cron RQ (2022). Therapeutic strategies for treating juvenile idiopathic arthritis. Curr Opin Pharmacol.

[4] Ringold S, Angeles Han ST, Beukelman T, Lovell D, Cuello CA, Becker ML (2019). 2019 American College of Rheumatology/Arthritis Foundation guideline for the treatment of juvenile idiopathic arthritis: therapeutic approaches for non systemic Polyarthritis, Sacroiliitis, and Enthesitis. Arthritis Care Res.

[5] Brunner HI, Ruperto N, Zuber Z, Keane C, Harari O, Kenwright A (2015). Efficacy and safety of tocilizumab in patients with polyarticular-course juvenile idiopathic arthritis: results from a phase 3, randomised, double-blind withdrawal trial. Ann Rheum Dis.

[6] O’Shea JJ, Holland SM, Staudt LM (2013). JAKs and STATs in immunity, immunodeficiency, and cancer. N Engl J Med.

[7] Tsilifis C, Freeman AF, Gennery AR (2021). STAT3 hyper-IgE syndrome—an update and unanswered questions. J Clin Immunol.

[8] Vogel TP, Leiding JW, Cooper MA, Forbes Satter LR (2023). STAT3 gain-of-function syndrome. Front Pediatr.

[9] Madhok R, Crilly A, Watson J, Capell HA (1993). Serum interleukin 6 levels in rheumatoid arthritis: correlations with clinical and laboratory indices of disease activity. Ann Rheum Dis.

[10] Robak T, Gladalska A, Stepień H, Robak E (1998). Serum levels of interleukin-6 type cytokines and soluble interleukin-6 receptor in patients with rheumatoid arthritis. Mediators Inflamm.

[11] Kang S, Tanaka T, Narazaki M, Kishimoto T (2019). Targeting interleukin-6 signaling in clinic. Immunity.

[12] Isomäki P, Junttila I, Vidqvist KL, Korpela M, Silvennoinen O (2015). The activity of JAK-STAT pathways in rheumatoid arthritis: constitutive activation of STAT3 correlates with interleukin 6 levels. Rheumatology.

[13] Anderson AE, Pratt AG, Sedhom MAK, Doran JP, Routledge C, Hargreaves B (2016). IL-6-driven STAT signalling in circulating CD4+ lymphocytes is a marker for early anticitrullinated peptide antibody-negative rheumatoid arthritis. Ann Rheum Dis.

[14] De Benedetti F, Robbioni P, Massa M, Viola S, Albani S, Martini A (1992). Serum interleukin-6 levels and joint involvement in polyarticular and pauciarticular juvenile chronic arthritis. Clin Exp Rheumatol.

[15] Yang XO, Panopoulos AD, Nurieva R, Chang SH, Wang D, Watowich SS (2007). STAT3 regulates cytokine-mediated generation of inflammatory helper T cells. J Biol Chem.

[16] Schnell A, Littman DR, Kuchroo VK (2023). TH17 cell heterogeneity and its role in tissue inflammation. Nat Immunol.

[17] Yang P, Qian FY, Zhang MF, Xu AL, Wang X, Jiang BP (2019). Th17 cell pathogenicity and plasticity in rheumatoid arthritis. J Leukoc Biol.

[18] Maggi L, Mazzoni A, Cimaz R, Liotta F, Annunziato F, Cosmi L (2019). Th17 and Th1 lymphocytes in oligoarticular juvenile idiopathic arthritis. Front Immunol.

[19] Kikuchi J, Hashizume M, Kaneko Y, Yoshimoto K, Nishina N, Takeuchi T (2015). Peripheral blood CD4+CD25+CD127low regulatory T cells are significantly increased by tocilizumab treatment in patients with rheumatoid arthritis: increase in regulatory T cells correlates with clinical response. Arthritis Res Ther.

[20] Meyer A, Wittekind PS, Kotschenreuther K, Schiller J, von Tresckow J, Haak TH (2021). Regulatory T cell frequencies in patients with rheumatoid arthritis are increased by conventional and biological DMARDs but not by JAK inhibitors. Ann Rheum Dis.

[21] Samson M, Audia S, Janikashvili N, Ciudad M, Trad M, Fraszczak J (2012). Brief Report: Inhibition of interleukin-6 function corrects Th17/Treg cell imbalance in patients with rheumatoid arthritis. Arthritis Rheum.

[22] Pesce B, Soto L, Sabugo F, Wurmann P, Cuchacovich M, López MN (2013). Effect of interleukin-6 receptor blockade on the balance between regulatory T cells and T helper type 17 cells in rheumatoid arthritis patients. Clin Exp Immunol.

[23] Milner JD, Vogel TP, Forbes L, Ma CA, Stray-Pedersen A, Niemela JE (2015). Early-onset lymphoproliferation and autoimmunity caused by germline STAT3 gain-of-function mutations. Blood.

[24] Petty RE, Southwood TR, Manners P, Baum J, Glass DN, Goldenberg J (2004). International League of Associations for Rheumatology classification of juvenile idiopathic arthritis: second revision, Edmonton, 2001. J Rheumatol.

[25] Wallace CA, Giannini EH, Huang B, Itert L, Ruperto N, Childhood Arthritis Rheumatology Research Alliance (CARRA) (2011). American College of Rheumatology provisional criteria for defining clinical inactive disease in select categories of juvenile idiopathic arthritis. Arthritis Care Res.

[26] Yasuda K, Takeuchi Y, Hirota K (2019). The pathogenicity of Th17 cells in autoimmune diseases. Semin Immunopathol.

[27] Burkett PR, Meyer zuHorste G, Kuchroo VK (2015). Pouring fuel on the fire: Th17 cells, the environment, and autoimmunity. J Clin Invest.

[28] Wang J, Zhao X, Wan YY (2023). Intricacies of TGF-β signaling in Treg and Th17 cell biology. Cell Mol Immunol.

[29] Lee G (2018). The balance of Th17 versus Treg cells in autoimmunity. Int J Mol Sci.

[30] Chougnet C, Hildeman D (2016). Helios—controller of Treg stability and function. Transl Cancer Res.

[31] Timperi E, Barnaba V (2021). CD39 regulation and functions in T cells. Int J Mol Sci.

[32] Da M, Chen L, Enk A, Ring S, Mahnke K (2022). The multifaceted actions of CD73 during development and suppressive actions of regulatory T cells. Front Immunol.

[33] Overton WR (1988). Modified histogram subtraction technique for analysis of flow cytometry data. Cytometry.

[34] Julé AM, Hoyt KJ, Wei K, Gutierrez-Arcelus M, Taylor ML, Ng J (2021). Th1 polarization defines the synovial fluid T cell compartment in oligoarticular juvenile idiopathic arthritis. JCI Insight.

[35] Moncrieffe H, Nistala K, Kamhieh Y, Evans J, Eddaoudi A, Eaton S (2010). High expression of the Ectonucleotidase CD39 on T cells from the inflamed site identifies two distinct populations, one regulatory and one memory T cell population. J Immunol.

[36] Wehrens EJ, Mijnheer G, Duurland CL, Klein M, Meerding J, van Loosdregt J (2011). Functional human regulatory T cells fail to control autoimmune inflammation due to PKB/c-akt hyperactivation in effector cells. Blood.

[37] Nistala K, Moncrieffe H, Newton KR, Varsani H, Hunter P, Wedderburn LR (2008). Interleukin-17–producing T cells are enriched in the joints of children with arthritis, but have a reciprocal relationship to regulatory T cell numbers. Arthritis Rheum.

[38] Stelmaszczyk-Emmel A, Jackowska T, Rutkowska-Sak L, Marusak-Banacka M, Wąsik M (2012). Identification, frequency, activation and function of CD4+ CD25highFoxP3+ regulatory T cells in children with juvenile idiopathic arthritis. Rheumatol Int.

[39] Sznurkowska K, Boćkowska M, Zieliński M, Plata-Nazar K, Trzonkowski P, Liberek A (2018). Peripheral regulatory T cells and anti-inflammatory cytokines in children with juvenile idiopathic arthritis. Acta Biochim Pol.

[40] Olivito B, Simonini G, Ciullini S, Moriondo M, Betti L, Gambineri E (2009). Th17 transcription factor RORC2 is inversely correlated with FOXP3 expression in the joints of children with juvenile idiopathic arthritis. J Rheumatol.

[41] Szymańska-Kałuża J, Cebula-Obrzut B, Smolewski P, Stanczyk J, Smolewska E (2014). Imbalance of Th17 and T-regulatory cells in peripheral blood and synovial fluid in treatment naïve children with juvenile idiopathic arthritis. Cent Eur J Immunol.

[42] DuPage M, Bluestone JA (2016). Harnessing the plasticity of CD4+ T cells to treat immune-mediated disease. Nat Rev Immunol.

[43] Qiu R, Zhou L, Ma Y, Zhou L, Liang T, Shi L (2020). Regulatory T cell plasticity and stability and autoimmune diseases. Clin Rev Allergy Immunol.

[44] Omoyinmi E, Hamaoui R, Pesenacker A, Nistala K, Moncrieffe H, Ursu S (2012). Th1 and Th17 cell subpopulations are enriched in the peripheral blood of patients with systemic juvenile idiopathic arthritis. Rheumatology.

[45] Holzer MT, Almanzar G, Woidich R, Hügle B, Haas JP, Prelog M (2022). Mitigated suppressive function of regulatory T cells (Treg) upon Th17-inducing cytokines in oligo- and polyarticular Juvenile Idiopathic Arthritis (JIA) patients. Pediatr Rheumatol.

[46] Wu SA, Yeh KW, Lee WI, Yao TC, Huang JL (2016). Persistent improper upregulation of Th17 and T Reg cells in patients with juvenile idiopathic arthritis. J Microbiol Immunol Infect.

[47] Cosmi L, Cimaz R, Maggi L, Santarlasci V, Capone M, Borriello F (2011). Evidence of the transient nature of the Th17 phenotype of CD4+CD161+ T cells in the synovial fluid of patients with juvenile idiopathic arthritis. Arthritis Rheum.

[48] Mahendra A, Misra R, Aggarwal A (2009). Th1 and Th17 predominance in the enthesitis-related arthritis form of juvenile idiopathic arthritis. J Rheumatol.

[49] Rosser EC, Lom H, Bending D, Duurland CL, Bajaj Elliott M, Wedderburn LR (2019). Innate lymphoid cells and T cells contribute to the interleukin 17A signature detected in the synovial fluid of patients with juvenile idiopathic arthritis. Arthritis Rheumatol.

[50] Patrick AE, Shoaff K, Esmond T, Patrick DM, Flaherty DK, Graham TB (2022). Increased development of Th1, Th17, and Th1.17 cells under T1 polarizing conditions in juvenile idiopathic arthritis. Front Immunol.

[51] Grose RH, Millard DJ, Mavrangelos C, Barry SC, Zola H, Nicholson IC (2012). Comparison of blood and synovial fluid Th17 and novel peptidase inhibitor 16 Treg cell subsets in juvenile idiopathic arthritis. J Rheumatol.

[52] Throm AA, Moncrieffe H, Orandi AB, Pingel JT, Geurs TL, Miller HL (2018). Identification of enhanced IFN-γ signaling in polyarticular juvenile idiopathic arthritis with mass cytometry. JCI Insight.

[53] Ptacek J, Hawtin RE, Sun D, Louie B, Evensen E, Mittleman BB (2021). Diminished cytokine-induced Jak/STAT signaling is associated with rheumatoid arthritis and disease activity. Plos One.

[54] Malle L, Martin-Fernandez M, Buta S, Richardson A, Bush D, Bogunovic D (2022). Excessive negative regulation of type I interferon disrupts viral control in individuals with down syndrome. Immunity.

[55] Nistala K, Adams S, Cambrook H, Ursu S, Olivito B, de Jager W (2010). Th17 plasticity in human autoimmune arthritis is driven by the inflammatory environment. Proc Natl Acad Sci.

[56] Maggi L, Cimaz R, Capone M, Santarlasci V, Querci V, Simonini G (2014). Brief report: Etanercept inhibits the tumor necrosis factor α–driven shift of Th17 lymphocytes toward a nonclassic Th1 phenotype in juvenile idiopathic arthritis. Arthritis Rheumatol.

[57] Onel KB, Horton DB, Lovell DJ, Shenoi S, Cuello CA, Angeles-Han ST (2022). 2021 American College of Rheumatology guideline for the treatment of juvenile idiopathic arthritis: therapeutic approaches for Oligoarthritis, Temporomandibular joint arthritis, and systemic juvenile idiopathic arthritis. Arthritis Care Res.

[58] Klein-Wieringa IR, Brinkman DMC, ten Cate R, Hissink Muller PCE (2020). Update on the treatment of nonsystemic juvenile idiopathic arthritis including treatment-to-target: is (drug-free) inactive disease already possible?. Curr Opin Rheumatol.

[59] Halyabar O, Mehta J, Ringold S, Rumsey DG, Horton DB (2019). Treatment withdrawal following remission in juvenile idiopathic arthritis: a systematic review of the literature. Pediatr Drugs.

[60] Gieling J, van den Bemt B, Hoppenreijs E, Schatorjé E (2022). Discontinuation of biologic DMARDs in non-systemic JIA patients: a scoping review of relapse rates and associated factors. Pediatr Rheumatol.

[61] Shoop-Worrall SJW, Kearsley-Fleet L, Thomson W, Verstappen SMM, Hyrich KL (2017). How common is remission in juvenile idiopathic arthritis: a systematic review. Semin Arthritis Rheum.

