# Supplementary material for: Th17/1 and ex-Th17 cells are detected in patients with polyarticular juvenile arthritis and increase following treatment
Source: Pediatr Rheumatol Online J. 2024 Mar 2;22:32. doi: 10.1186/s12969-024-00965-5 (PMC10908086; doi:10.1186/s12969-024-00965-5)
Supplement: Supplementary file 1 — Supplementary Materials 1. [file 12969_2024_965_MOESM1_ESM.pdf]

Supplemental Figure 1

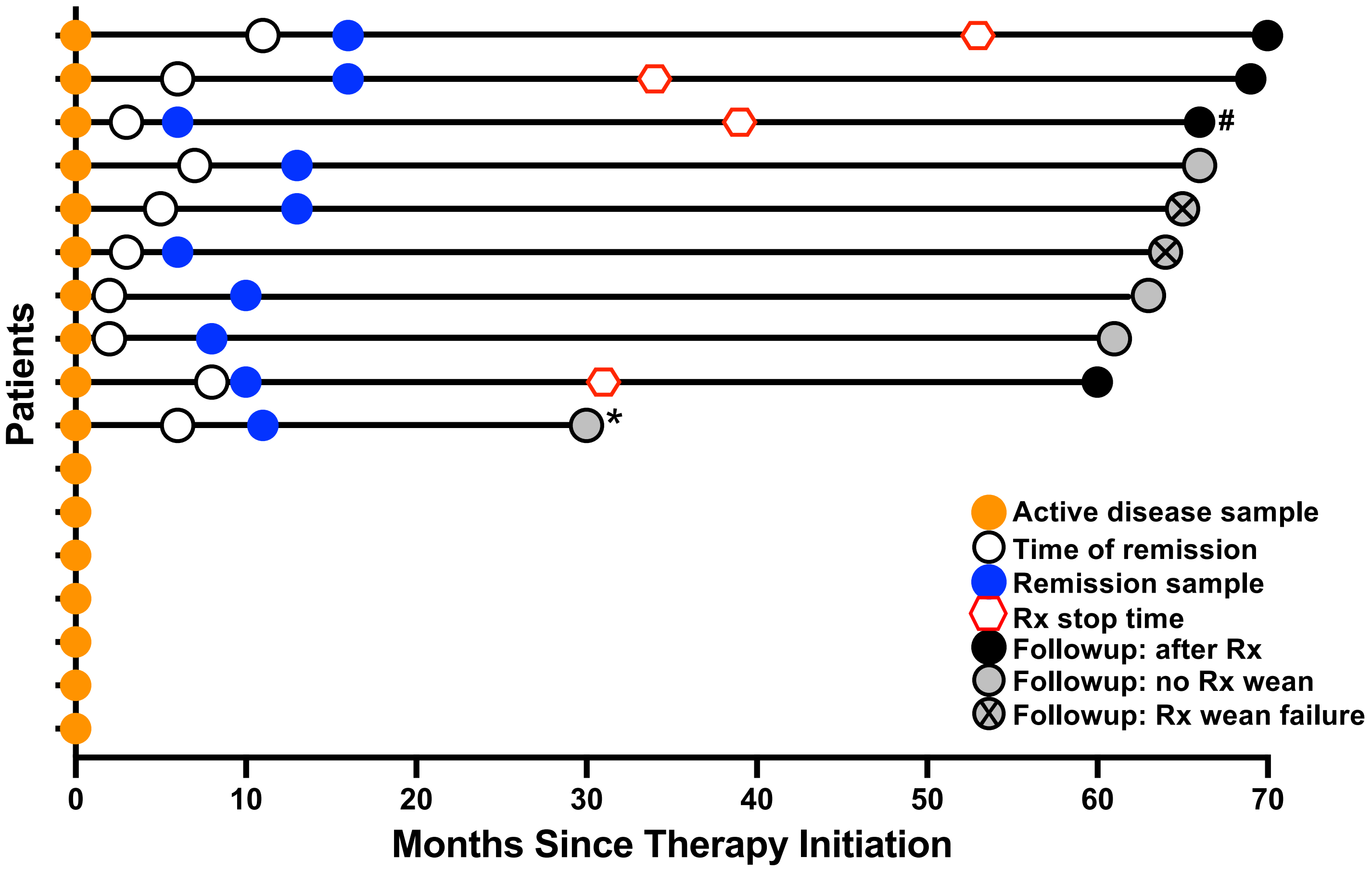

Patient sample collection timeline. PBMCs were collected from treatment-naïve patients with active disease (orange) at time of diagnosis and treatment (Rx) initiation (0 months). Subsequent samples were collected after remission (white) when possible (blue, post-treatment). Treatment was successfully discontinued in 4 patients (red), who have been free of active disease for years of followup (black). Medications have not yet been weaned in 4 patients (gray), one of whom developed refractory uveitis. For 2 patients (gray "x"), multiple attempts to wean medications have been unsuccessful. #, patient treated with tocilizumab monotherapy (all other patients with matched samples who received a biologic received a tumor necrosis factor (TNF)α inhibitor); *, patient censored at time of last followup (patient moved); PBMC, peripheral blood mononuclear cells

Supplemental Figure 2

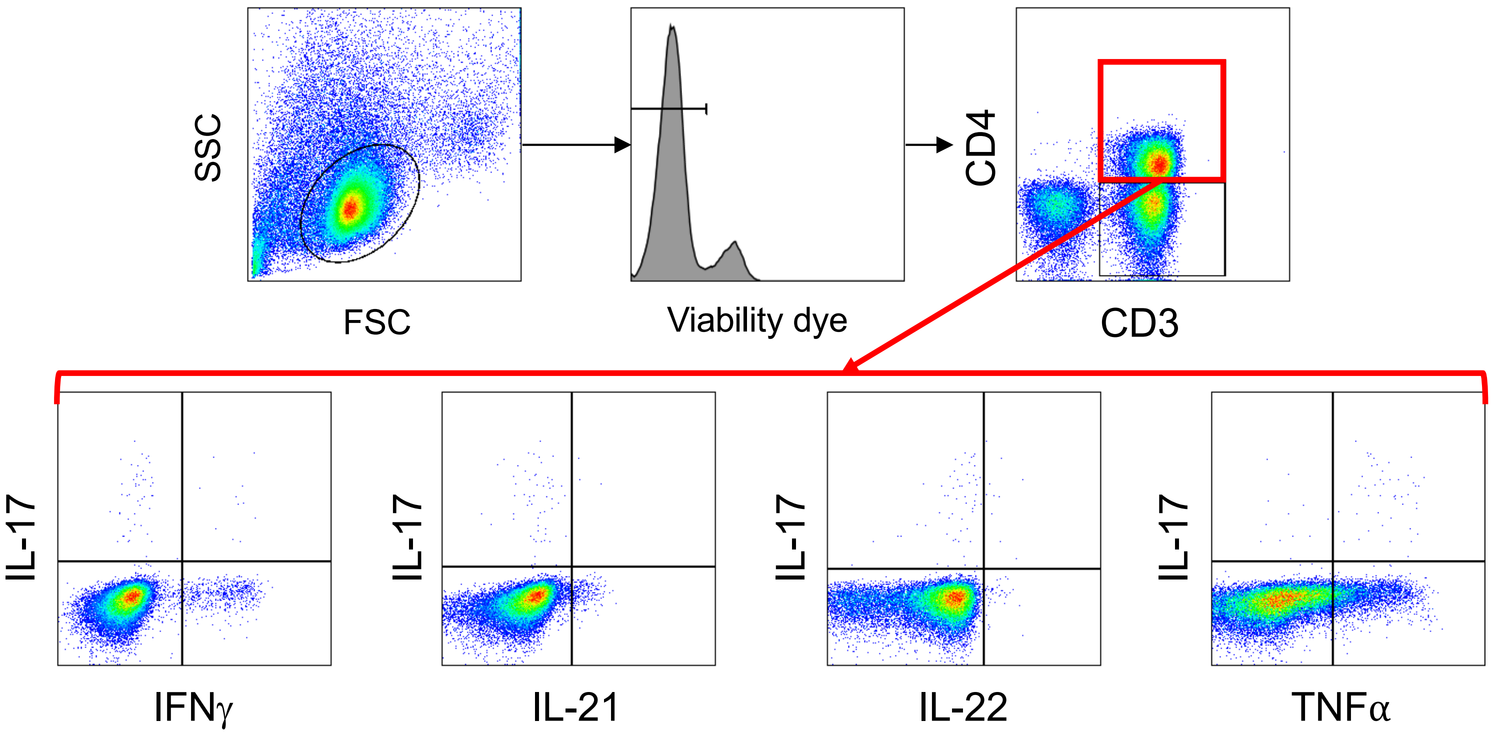

Gating strategy for Th17 cell flow analysis. Representative analysis of PBMCs from a healthy control after overnight stimulation with PMA+calcimycin. Sequentially: Lymphocytes were identified by size and granularity, followed by live cell discrimination using a viability dye, and then CD4+ T cells (CD3+), which downregulate CD4 with stimulation, were assessed for cytokine production: interleukin (IL)-17, interferon (IFN)γ, IL-21, IL-22, and tumor necrosis factor (TNF)α.

Supplemental Figure 3

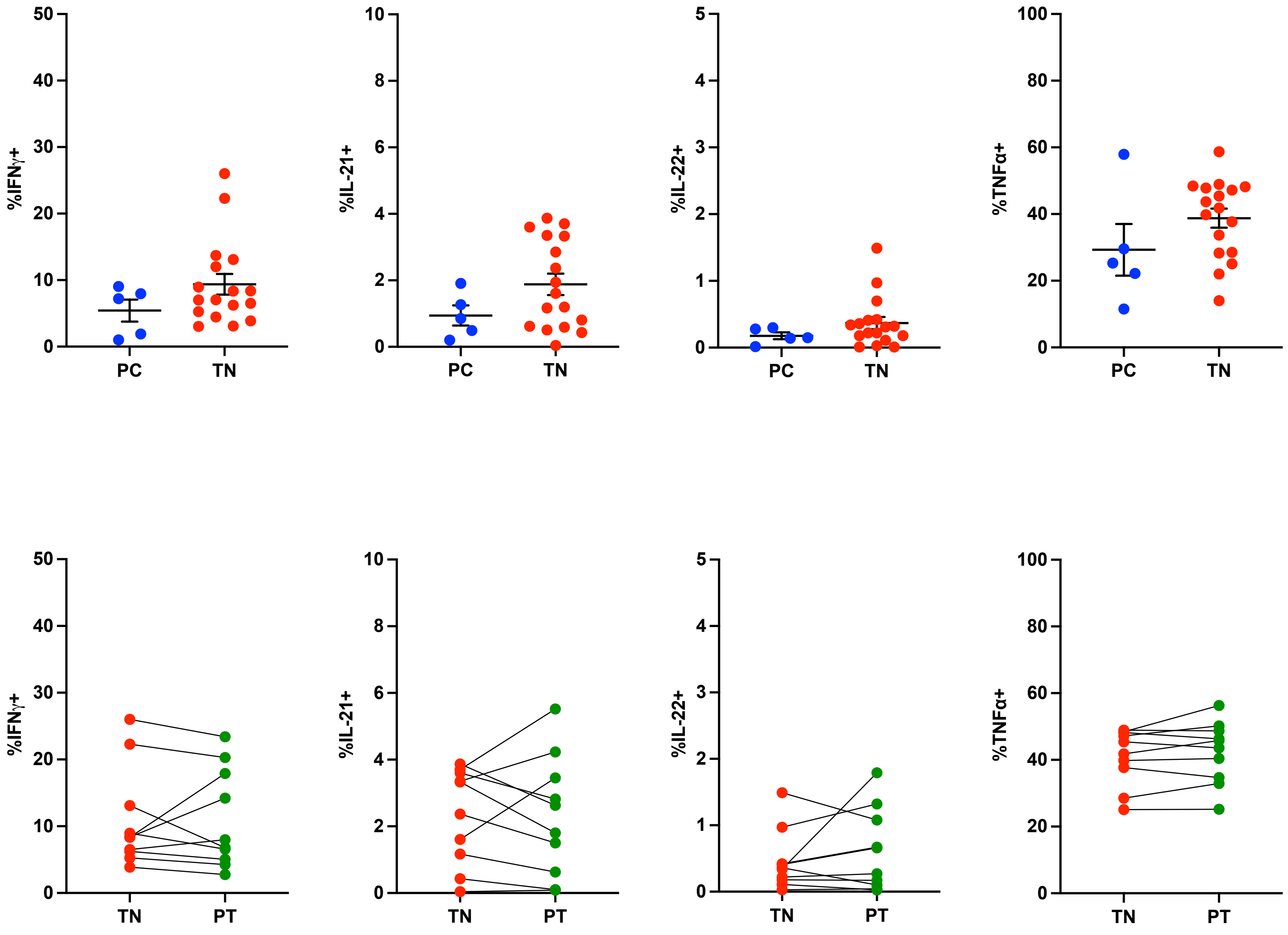

Inflammatory cytokine production trends higher in CD4+ T cells from polyJIA patients. Top row: PBMCs from healthy pediatric controls (PC) and treatment-naïve polyJIA patients (TN) were analyzed for CD3+CD4+ T cells producing proinflammatory cytokines after overnight stimulation with PMA+calcimycin (Mann-Whitney U test). Bottom row: Samples from polyJIA patients that had achieved remission on medication post-treatment (PT) were also analyzed and compared to the TN frequencies for that same patient (Wilcoxon matched-pairs signed rank test). Analysis did not reveal significant differences, although proinflammatory cytokine production by CD4+ T cells from TN polyJIA patients trended higher compared to PC. Black bars represent mean ± SEM. PBMCs, peripheral blood mononuclear cells; PMA, phorbol 12-myristate 13-acetate; IFN, interferon, IL, interleukin; TNF, tumor necrosis factor

Supplemental Figure 4

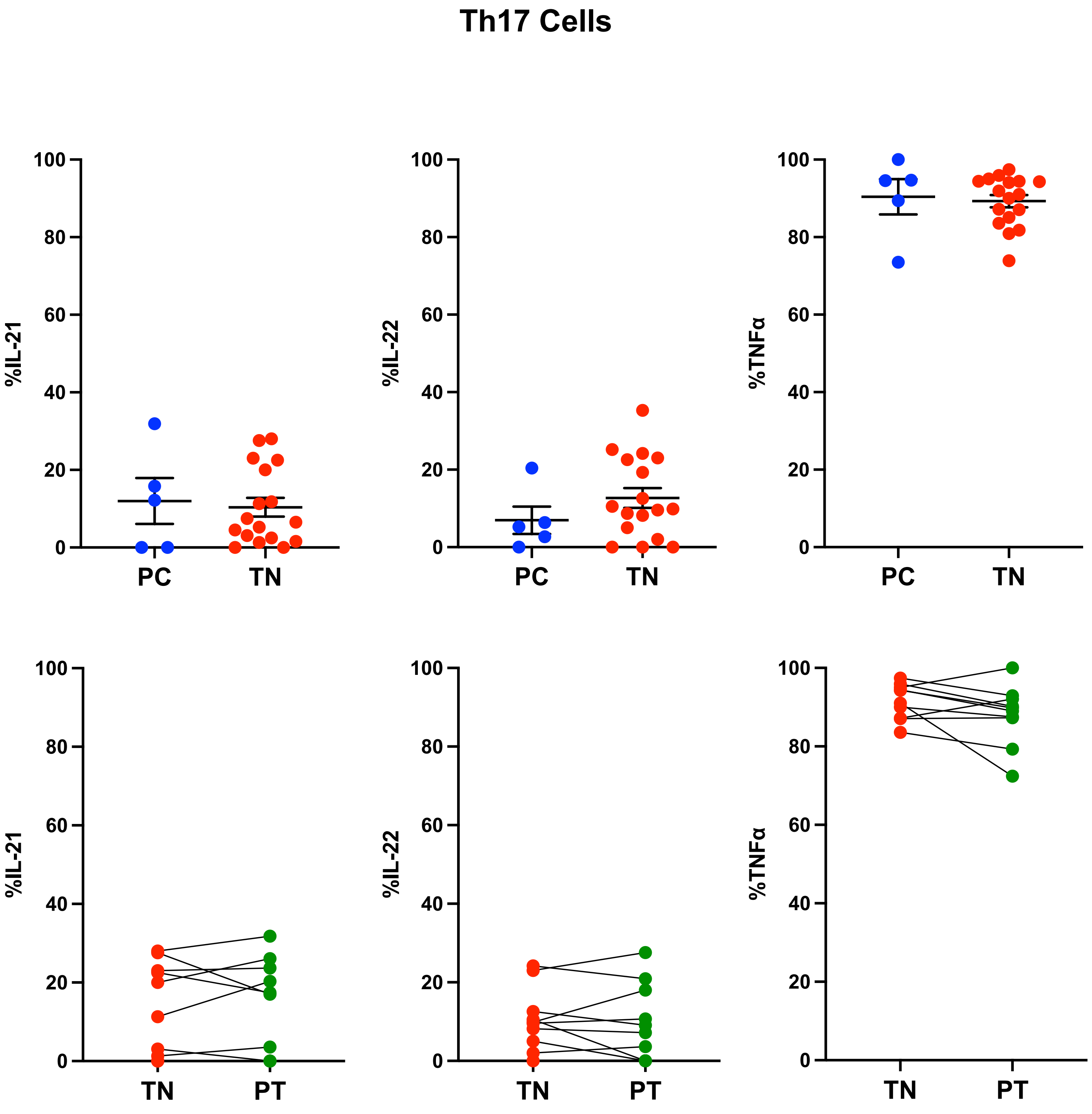

Th17 cells from pediatric controls and polyJIA patients produce proinflammatory cytokines at similar frequencies. Top row: PBMCs from healthy pediatric controls (PC) and treatment-naïve polyJIA patients (TN) were analyzed for the frequencies of CD3+CD4+IL-17+ (Th17) cells producing proinflammatory Th17-associated cytokines after overnight stimulation with PMA+calcimycin (Mann-Whitney U test). Bottom row: Samples from polyJIA patients that had achieved remission on medication post-treatment (PT) were also analyzed and compared to the TN frequencies for that same patient (Wilcoxon matched-pairs signed rank test). Analysis did not reveal significant differences. Black bars represent mean ± SEM. PBMCs, peripheral blood mononuclear cells; PMA, phorbol 12-myristate 13-acetate; IL, interleukin; TNF, tumor necrosis factor

Supplemental Figure 5

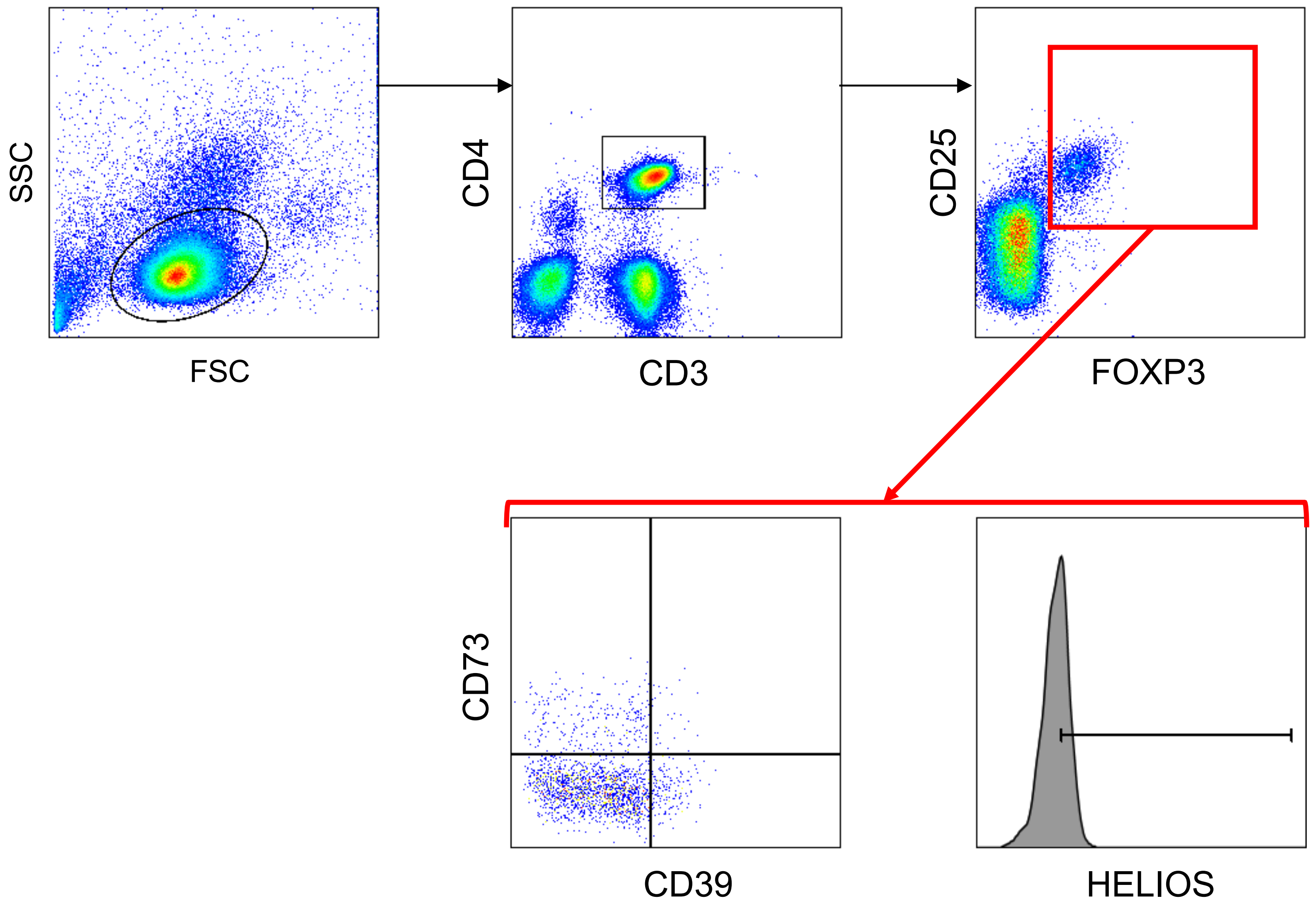

Gating strategy for Treg cell flow analysis. Representative *ex vivo* analysis of PBMCs from a healthy control. Sequentially: Lymphocytes were identified by size and granularity, then CD4+ T cells (CD3+) were assessed for CD25 and FOXP3 dual expression to identify Tregs, which were further analyzed for CD73, CD39, and HELIOS expression. CD25, FOXP3, CD73, CD39 and HELOIS gates were set using isotype control antibodies.

Supplemental Figure 6

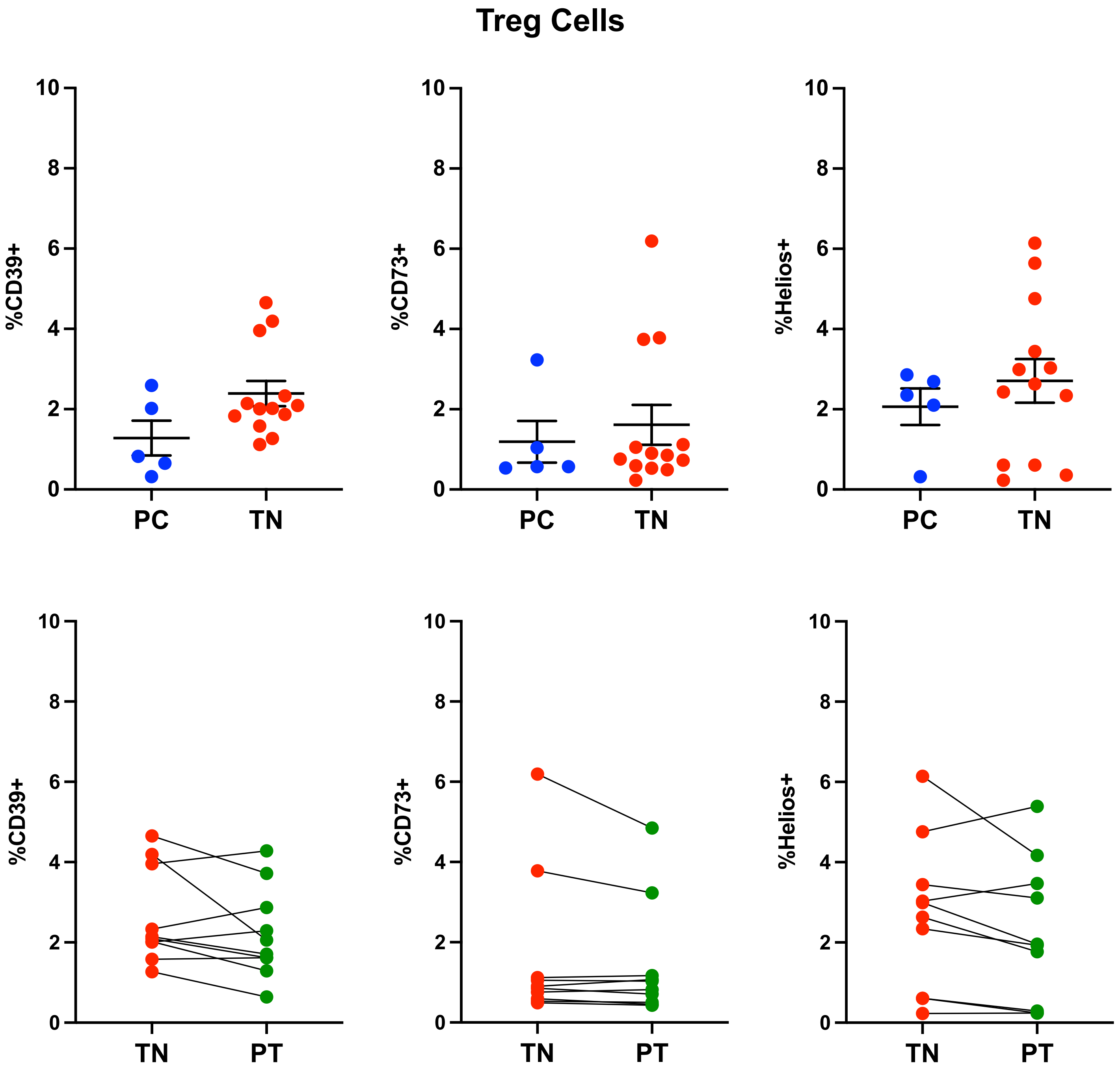

Treg cells from some polyJIA patients express activation markers at higher frequencies. Top row: PBMCs from healthy pediatric controls (PC) and treatment-naïve polyJIA patients (TN) were analyzed *ex vivo* for the frequencies of CD3+CD4+CD25+FOXP3+ (Treg) cells also expressing CD39, CD73, or HELIOS (Mann-Whitney U test). Bottom row: Samples from polyJIA patients that had achieved remission on medication post-treatment (PT) were also analyzed and compared to the TN frequencies for that same patient (Wilcoxon matched-pairs signed rank test). Analysis did not reveal significant differences. Black bars represent mean ± SEM.

Supplemental Figure 7

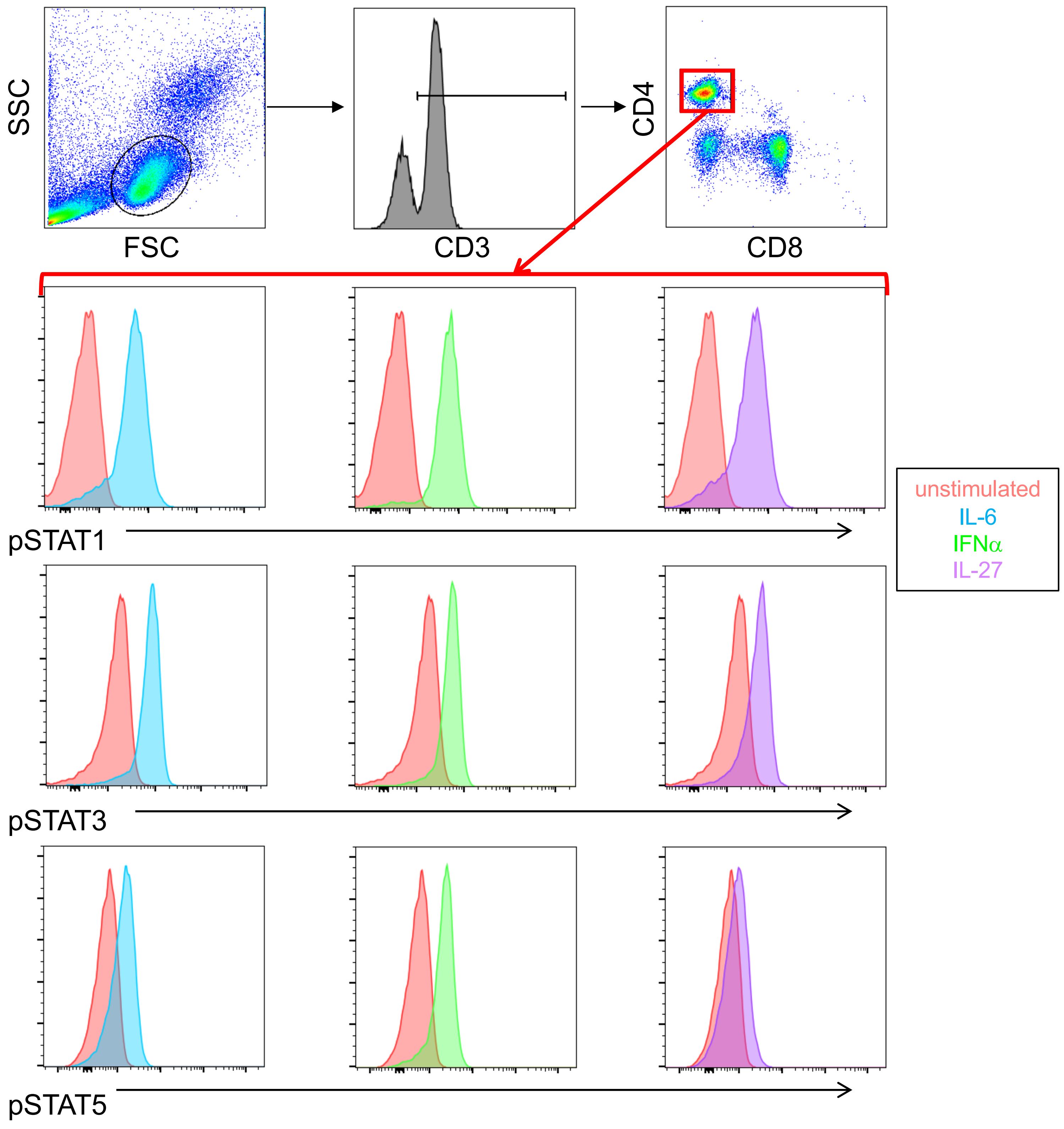

Gating strategy for phospho-STAT flow analysis. Representative analysis of PBMCs from a healthy control. PBMCs were left unstimulated (pink) or stimulated *ex vivo* for 30 minutes with IL-6 (blue), IFNα (green), or IL-27 (purple). Sequentially: Lymphocytes were identified by size and granularity, then CD3+ T cells were assessed for major subsets. Activated/phosphorylated (p)STATs 1, 3, and 5 were assessed in CD4+ T cells. PBMCs, peripheral blood mononuclear cells; IL, interleukin; IFN, interferon

Supplemental Figure 8

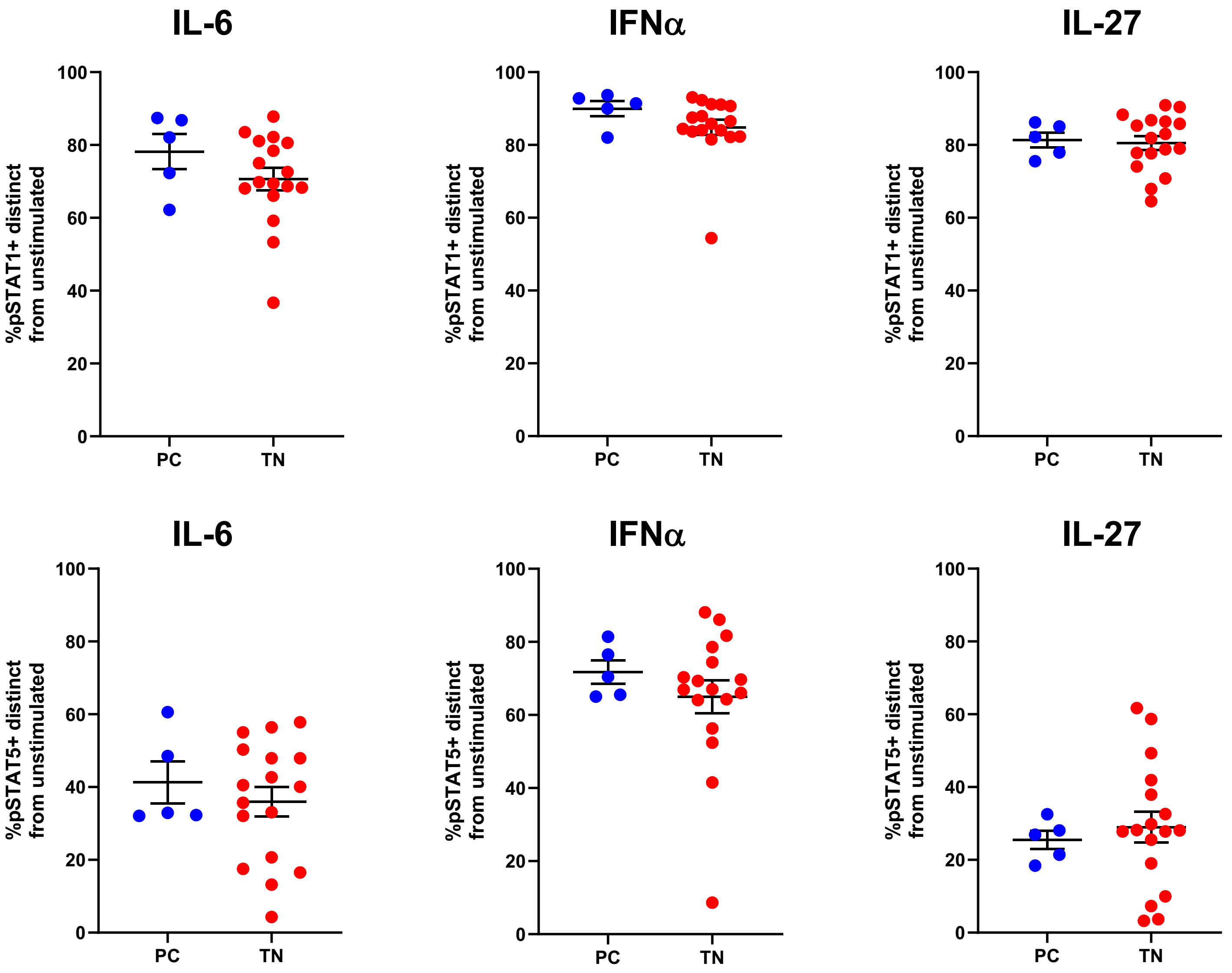

CD4+ T cell responses to inflammatory cytokine stimulation *ex vivo*. PBMCs were left unstimulated or stimulated *ex vivo* for 30 minutes with IL-6, IFNα, or IL-27, then activated/phosphorylated (p)STAT1 and pSTAT5 measured using flow cytometry. Data represent the %pSTAT+ cells in a stimulated sample distinct from when unstimulated, generated using Overton subtraction. There were no significant differences between cells from treatment-naïve (TN) patients and those from healthy pediatric controls (PC) (Mann-Whitney U test). Black bars represent mean ± SEM. PBMCs, peripheral blood mononuclear cells; IL, interleukin; IFN, interferon

Supplemental Figure 9

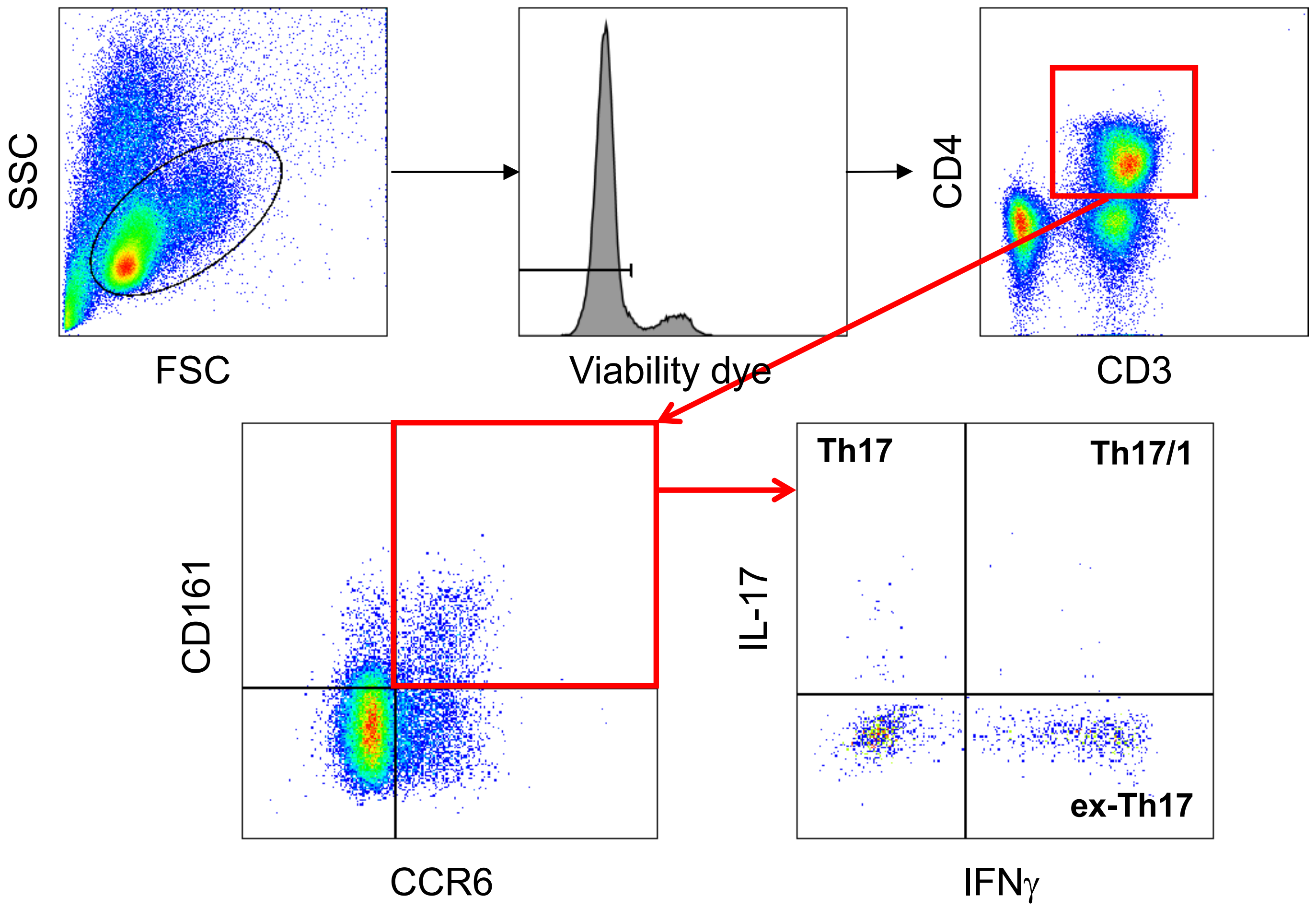

Gating Strategy for T cell plasticity flow analysis. Representative analysis of PBMCs from a polyJIA patient after overnight stimulation with PMA+calcimycin. Sequentially: Lymphocytes were identified by size and granularity, followed by live cell discrimination using a viability dye, and then CD4+ T cells (CD3+), which can downregulate CD4 with stimulation, were assessed further for CD161, CCR6, and cytokine production: interleukin (IL)-17 and interferon (IFN)γ. Cytokine expressing subsets identified are Th17 (CCR6+CD161+IL-17+), Th17/1 (CCR6+CD161+IL-17+IFNγ+), and ex-Th17 (CCR6+CD161+IFNγ+) cells.
