# Supplementary material for: Th17/1 and ex-Th17 cells are detected in patients with polyarticular juvenile arthritis and increase following treatment
Source: Pediatr Rheumatol Online J. 2024 Mar 2;22:32. doi: 10.1186/s12969-024-00965-5 (PMC10908086; doi:10.1186/s12969-024-00965-5)
Supplement: Supplementary file 2 — Supplementary Materials 2. [file 12969_2024_965_MOESM2_ESM.pdf]

Supplemental Methods: Antibodies

Th17 Panel

Marker	Fluorophore	Clone	Company	Catalog#
IL-17	FITC	N49-653	BD	560488
IFNγ	PE	B27	BD	559327
CD4	PerCP	RPA-T4	BD	560650
IL-22	PECy7	2G12A41	Biolegend	366708
IL-21	APC	3A3-N21	BD	560493
CD3	APCCy7	SK7	BD	557832
TNFα	BV421	MAB11	BD	562783
Live/Dead	Zombie Yellow	-	Biolegend	423104

Treg Panel

Marker	Flurophore	Clone	Company	Catalog#	Isotype Clone	Company	Catalog#
HELIOS	FITC	22F6	Invitrogen	11-9883-80	eBio299ARM	eBiosciences	11-4888-81
FOXP3	PE	206D	Biolegend	320108	MOPC-21	Biolegend	400140
CD4	PerCP	RPA-T4	BD	560650	-	-	-
CD73	PECy7	AD2	BD	561258	MOPC-21	BD	557872
CD25	APC	BC96	Biolegend	302610	eBM2a	eBiosciences	50-4724-80
CD39	APCCy7	A1	Biolegend	328225	MOPC-21	Biolegend	400128
CD3	BV510	UCHT1	BD	563109	-	-	-

STAT Panel

Antibodies	Flurophore	Clone	Company	Catalog#
pSTAT3 Y705	PE	4/P-STAT3	BD	562072
CD4	PECy7	SK3	BD	557852
pSTAT1 Y701	APC	4a	BD	562070
CD3	APCCy7	SK7	BD	557832
pSTAT5 Y694	BV421	47/Stat5(pY694)	BD	562984
CD8	BV510	RPA-T8	BD	563256

Th17/1 and ex-Th17 Panel

Antibodies	Flurophore	Clone	Company	Catalog#	Isotype Clone	Company	Catalog#
IL-17	FITC	N49-653	BD	560488	N49-653	BD	560488
IFNγ	PE	B27	BD	559327	B27	BD	559327
CD4	PerCP	RPA-T4	BD	560650	-	-	-
CD161	APC	DX12	BD	550968	DX12	BD	550968
CD3	APCCy7	SK7	BD	557832	-	-	-
CCR6	BV421	G034E3	Biolegend	353408	G034E3	Biolegend	353408
Live/Dead	Zombie Yellow	-	Biolegend	423104	-	-	-
